# Imperforate Hymen - a rare cause of acute abdominal pain and tenesmus: case report and review of the literature

**DOI:** 10.11604/pamj.2013.15.28.2251

**Published:** 2013-05-21

**Authors:** Aruyaru Stanley Mwenda

**Affiliations:** 1 Transmara District Hospital-Kilgoris, Kenya

**Keywords:** Imperforate hymen, amenorrhea, pubertal girls, urine retention

## Abstract

Imperforate hymen is a rare condition that presents with amenorrhea, cyclical abdominal pains and urine retention among pubertal girls. A 14 year old girl with imperforate hymen underwent hymenotomy for hematocolpometra, having presented with abdominal pains and tenesmus.

## Introduction

Imperforate hymen, despite being the commonest female genital tract malformation [1], is a rare occurrence with a prevalence of 0.014-0.1% [1–3]. It mostly presents during puberty [1, 4] although diagnoses in utero [3, 5, 6] and during the new born period and childhood [3, 7] are also documented.

There are few cases of Imperforate hymen reported in Africa. A case of unique presentation with tenesmus besides other documented symptoms was managed at a rural Kenyan hospital. There is no recorded case of imperforate hymen presenting with tenesmus according to literature search. In this article, a review of the literature concerning the symptomatology of imperforate hymen among pubertal girls is also presented.

## Patient and observation

14 year old Kenyan girl of African descent presented to hospital with a weeklong complaint of lower abdominal pains associated with tenesmus. She had reduced appetite and poor intake of food due to the colicky pains. There was no abdominal distension but she had observed some suprapubic fullness. She did not have constipation, diarrhoea, vomiting or fevers. Her urinary habits were normal. She had never had her menstrual periods but she had developed secondary sexual characteristics.

**Figure 1:**
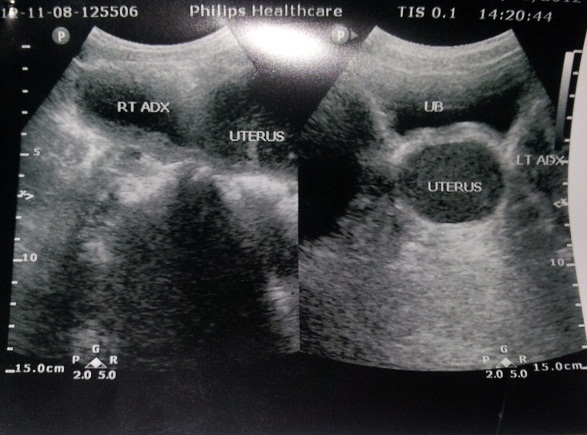
Distended uterus

**Figure 2:**
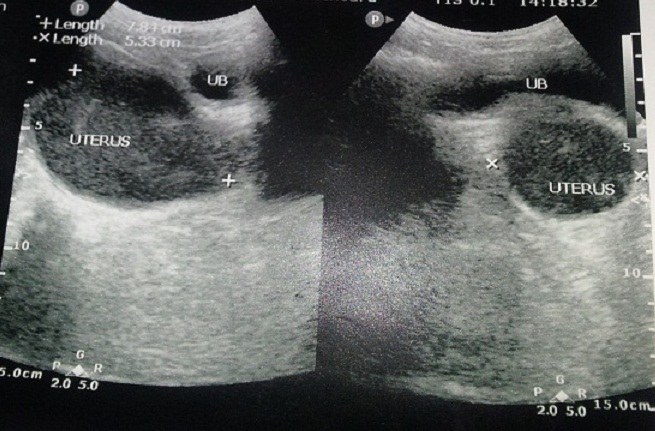
Bladder compression by the distended uterus

On examination, she was in severe pain, walking stooped over and had moderately tender suprapubic mass corresponding to a uterus at 16 weeks. Rectal examination revealed an anterior mass. Perineal examination revealed a bulging imperforate hymen exaggerated on valsava manoeuvre. Pelvic ultrasound done revealed distended uterus and vagina all filled up with homogenous thick fluid (Figure 1, Figure 2). A diagnosis of hematocolpometra was made.

In theatre, an X-shaped incision of the hymen was made under anaesthesia and approximately 600mls of thick chocolate coloured blood evacuated. The edges of the hymen were everted and anchored by Vicryl 2/0 sutures. Analgesic cream and prophylactic oral antibiotics were prescribed. She made uneventful recovery and was doing well at 1 month. She was however lost to follow-up after that.

## Discussion

Imperforate hymen is a layer of connective tissue that forms a transverse septum and obstructs the vaginal opening at the level of the introitus [5]. Usually, the hymen is a membrane that embryologically develops through the fusion of the caudal end of the paramesonephric ducts and the urogenital sinus [4, 5, 7, 8]. The central portion of this membrane perforates through the degeneration of its epithelial cells [5]. Failure of the degeneration of the epithelial cells and subsequent perforation leads to a hymen that is termed imperforate [5].

The function of the hymen is not clear but is thought to include innate immunity as it provides a physical barrier to infections during the pre-pubertal period when the vaginal immunity is not fully developed [3].

Imperforate hymen is rarely associated with other female genital tract malformations [1, 4] although some authors [2, 9] have emphasized the need to rule out associated mullerian malformations. It occurs sporadically but few familial cases have been reported [8].

Imperforate hymen can present during three main stages in life;

1. In utero: This is the rarest and occurs due to maternal estrogenic stimulation that leads to uterovaginal secretions filling up the blind vagina and presenting as hydrocolpos diagnosed through obstetric ultrasound [6]. The diagnosis should be confirmed post natally.
2. New-born-infanthood-childhood: In new-born period this may occur due to maternal estrogenic stimulation that leads to uterovaginal secretions filling up the blind vagina and presenting with hydrocolpos [1, 3, 7, 8, 10].
3. At puberty: This is the commonest. It occurs when a girl starts menstruating and the menstrual blood accumulates in the vagina [3, 10]. The age of presentation (mean, range) is 13.2 and 11-16 years respectively according to Liang et al [5] or 12 and 10-15 years respectively according to Lui et al [9]. Liang and colleagues did a ten year retrospective analysis of 15 women treated for imperforate hymen through telephone based researcher administered questionnaire and a subsequent physical and sonographic examination. In their study, Lui et al did a ten year retrospective analysis of the data of 15 patients treated for imperforate hymen but did not do any follow up patient interview or examination. Kurgodlu and colleagues argue that the age of presentation is 2.5-4 years after thelarche [12].

Among the pubertal girls, imperforate hymen will present in the following ways.

### Amenorrhea

- Primary amenorrhea
  - This is because the girl has started menstruating but does not experience any menstrual flow as the blood accumulates in the vagina, then in the uterus and occasionally, eventually into the fallopian tubes [3, 4, 7].
- Secondary amenorrhea
  - This can occur following spontaneous closure of previously perforate hymen [8]. This can happen with a micro perforate or stenosed hymen. In such initial light periods will be experienced but continuous stenosis leads to complete obstruction and amenorrhea [8].
  - It can also occur as a result of stenosis of the hymenal opening following surgical or sexual trauma [8].
  - Lastly, it can occur as failure of hymenotomy [10]. In the months following hymenotomy the patient experiences her menstrual flow but the margins of the hymenotomy incision adhere and eventually occlude the vaginal outflow leading to amenorrhea.
- Cryptomenorrhea

### Pain

Recurrent cyclical lower abdominal/pelvic pains (up to 60%) [2, 4, 8, 9, 11, 12]. This is due to continued distension of the vagina and uterus by accumulating menstrual blood.

Low back pain (38-40%) [4, 13, 14]. Occurs as referred pain following irritation of the sacral plexus and nerve roots by the distended vagina and uterus.

### Obstruction

- Urinary outflow obstruction and its complications (58%) [09]
  - Acute urine retention (3-60%) [7, 09, 10, 13, 15].This occurs by a number of mechanisms
    - Pressure on the bladder by the distended uterus causing angulation at the bladder neck and kinking of the urethra [10]
    - Direct pressure on the urethra causing urethral tamponade [10]
    - The bulging hymen distends the vagina and may cause cephalad angulation at the urethral meatus further stretching the urethra and worsening tamponade [15].
  - Complications of prolonged or recurrent urine retention /obstruction
    - Hydroureters [2]
    - Hydronephrosis [2]
    - Renal failure [1]
    - Acute bacterial nephritis [16]
- Vaginal outflow obstruction- Cryptomenorrhea
- Intestinal obstruction
  - Constipation (20-27%) [9, 13]
  - Tenesmus
- Lymphovenous obstruction

Compression of the pelvic veins and lymphatics can impair lymphovenous return from the lower limbs leading to oedema [1].

### Mass

- Distended uterus felt as pelvic mass on abdominal examination (20%) [9]
- The distended vagina is felt as a pelvic mass on digital rectal examination
- A bluish bulging hymen is observed beneath the labia (60%) [9]
- A cystic retropubic mass is revealed on ultra sonography or MRI [9]

With above in mind and a high index of suspicion, it is easy to make a diagnosis of imperforate hymen. Late presentation may be accompanied with complications such as ruptured hematosalpinx [9, 11], endometriosis [4, 15] and infection (pyocolpos and nephritis) [5, 16]. A clinical diagnosis negates the need for extensive laboratory and radiological investigations [10] and reduces the delay of intervention and length of hospital stay [9].

The management is aimed at re-establishing vaginal outflow and mainly consists of surgical hymenotomy under local or general anaesthesia [7]. Simple vertical, T-shaped, cruciform, X-shaped and cyclical incisions may be used [4, 7, 8]. X-shaped incision has the advantage of reduced risk of injury to the urethra-which should be stented during the procedure [7]. Pressure on the uterus in order to expel more blood is discouraged as it can lead to retrograde flow through the tubes causing endometriosis and tubal adhesions [15]. Hymenectomy and hymenotomy with a two week indwelling catheter have also been reported [8]. The outcome is good and the recurrences are rare [5].

## Conclusion

Imperforate hymen is a rare condition but should be easy to diagnose when it presents. It should be suspected in pubertal girls who presented with acute abdominal pain.
